# Fetus in fetu: two case reports and literature review

**DOI:** 10.1186/1471-2431-14-88

**Published:** 2014-04-02

**Authors:** Yi Ji, Siyuan Chen, Lin Zhong, Xiaoping Jiang, Shuguang Jin, Feiteng Kong, Qi Wang, Caihong Li, Bo Xiang

**Affiliations:** 1 Division of Oncology, Department of Pediatric Surgery, West China Hospital of Sichuan University, 37# Guo-Xue-Xiang, Chengdu 610041, China; 2 Pediatric Intensive Care Unit, West China Hospital of Sichuan University, Chengdu 610041, China; 3 Department of Pathology, West China Hospital of Sichuan University, Chengdu 610041, China

**Keywords:** Fetus in fetu, Teratoma, Preoperative diagnosis, Treatment

## Abstract

**Background:**

Fetus in fetu is a rare congenital anomaly and is defined as a monozygotic twin incorporated into the abdomen of its sibling during development. Fetus in fetu is often overlooked in the differential diagnosis of an abdominal mass. Unlike teratomas, fetus in fetu is a benign disorder.

**Case presentation:**

We describe the clinical characteristics of two patients, a thirty-months old boy who was found to have abdominal distension and a neonate who was diagnosed antenatally with abdominal mass. Computed tomography scan revealed the mass in which the contents favor a fetus in fetu rather than a teratoma. Surgical removal revealed that the anencephalic fetus have limb buds situated relative to a palpable vertebral column, supporting the diagnosis of fetus in fetu. In the present report, presentation, diagnosis, pathology, management, and recent literature are also reviewed.

**Conclusion:**

Fetus in fetu is a rare entity that typically presents in infancy and early childhood. It should be differentiated from a teratoma because of the teratoma’s malignant potential. Preoperative diagnosis is based on radiologic findings. The treatment of fetus in fetu is operative to relieve obstruction, prevent further compression and possible complications. Complete excision allows confirmation of the diagnosis and lowers the risk of recurrence.

## Background

Fetus in fetu (FIF) is a rare cause of abdominal mass in children. The pathogenesis of FIF can be explained by the ‘included-twin’ theory in which the FIF is a diamniotic, monochorionic, monozygotic twin that becomes incorporated into the body of the host twin after anastomosis of the vitelline circulation [1]. Although the most common site is the retroperitoneum, FIF have been reported at various sites right from the cranial cavity to the scrotal sac [2]. Different organs can be seen in FIF, including vertebral column (91%), limbs (82.5%), central nervous system (55.8%), gastrointestinal tract (45%), vessels (40%), and genitourinary tract (26.5%) [3]. It is differentiated from teratoma by the presence of vertebral organization with limb buds and other organ systems. A presumptive diagnosis can be made by ultrasonography, plain radiography, computed tomography, or magnetic resonance imaging.

## Case reports

### Case presentation N.1

**Figure 1:**
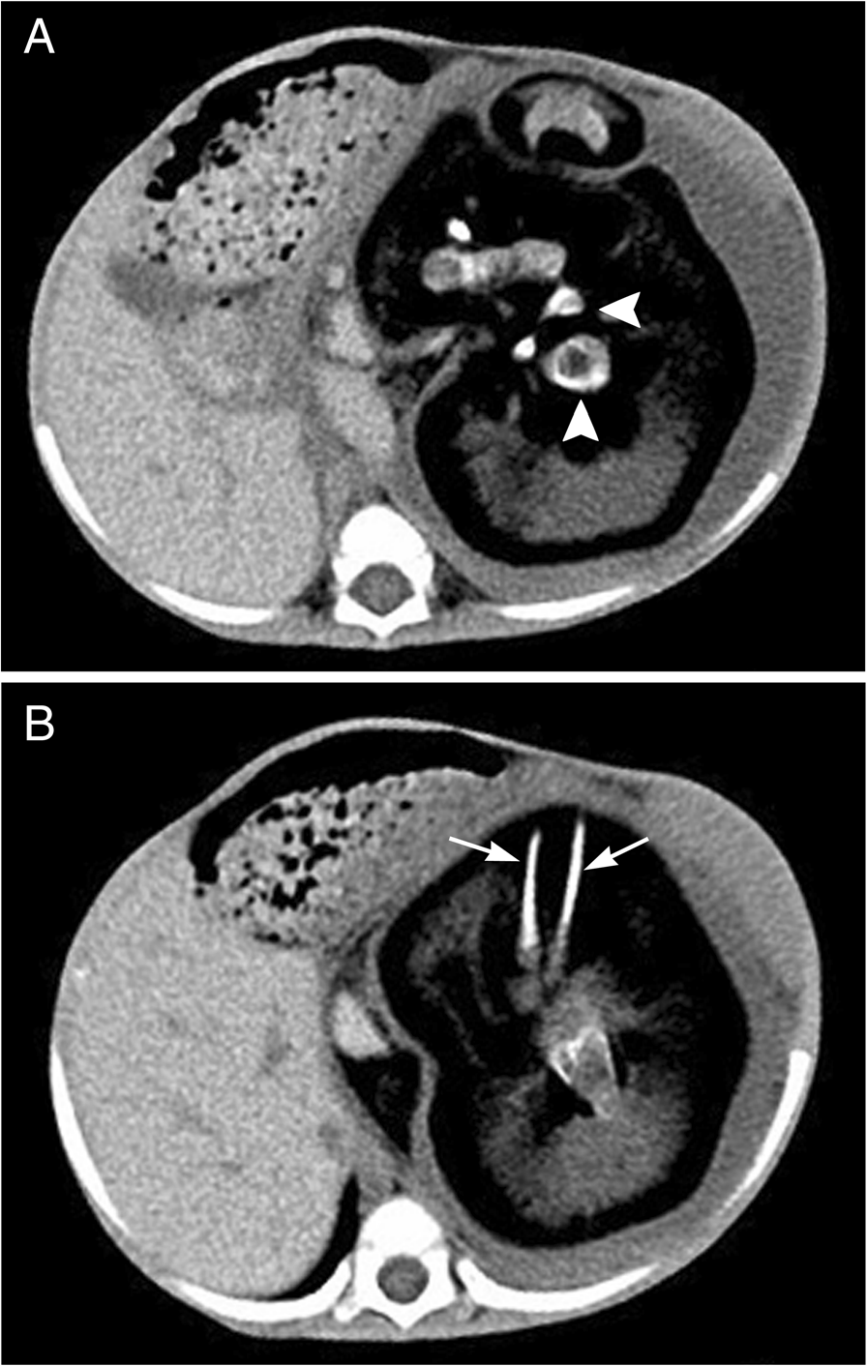
**Abdominal computed tomography shows a semisolid mass with calcified and lipomatous components within the cyst. A**. A well-formed vertebral body (Arrowhead); **B**. Arrow head shows the long opacities corresponding to fetal limbs.

A thirty months old boy was hospitalized because of an abdominal distension of 1 year’s duration. The boy started vomiting 2 months ago but continued to pass normal stools. He also had at least a 1-week history of respiratory distress. The boy was born full term by normal vaginal delivery with a birth weight of 3100 g. There was no family history of twins, and the remainder of the family and prenatal history was unremarkable. On abdominal examination there was a well defined firm, round, non-tender mass in the left upper abdomen. Complete blood count and kidney-liver function tests were within reference ranges. Both serum *β*-human chorionic gonadotropin (*β*-HCG) and serum α-fetoprotein (AFP) levels were normal. Serum carcinoembryonic antigen (CEA) level was 52.86 ng/mL (upper limit of normal 4.6 ng/mL). Computed tomography scan of the abdomen showed a large, complex, retroperitoneal mass with solid, cystic, and calcified elements. The calcifications in the mass had the appearance of vertebral body (Figure 1A) and lower extremity long bones (Figure 1B, arrow). The mass displaced the left kidney anteriorly. The boy received a diagnosis of FIF.

**Figure 2:**
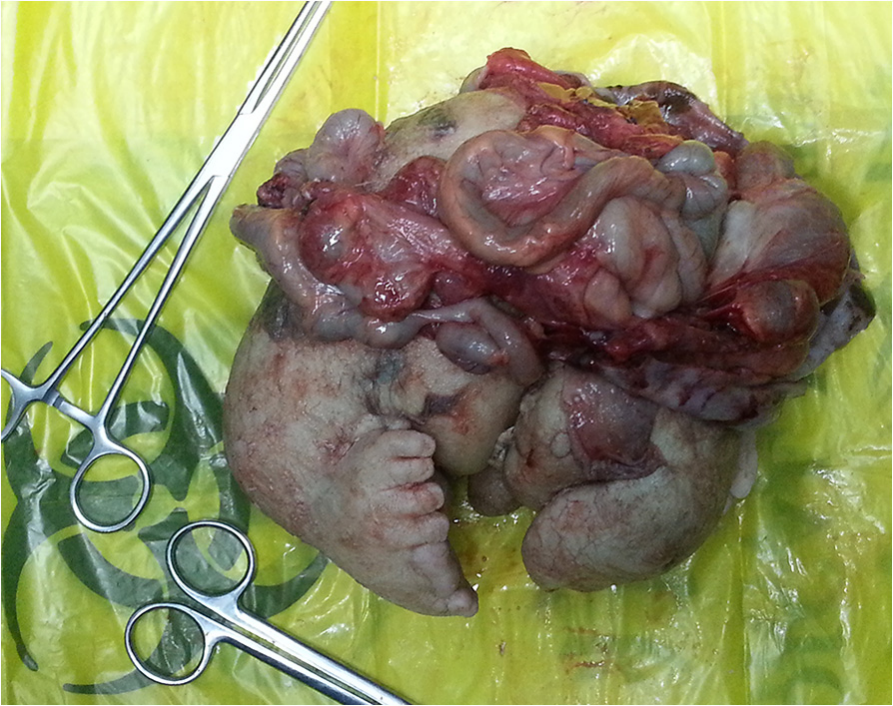
**The postoperative specimen shows a fairly well-developed fetus lying on its back.** The fetus is partially covered with skin, demonstrating intestines and two lower limbs.

**Figure 3:**
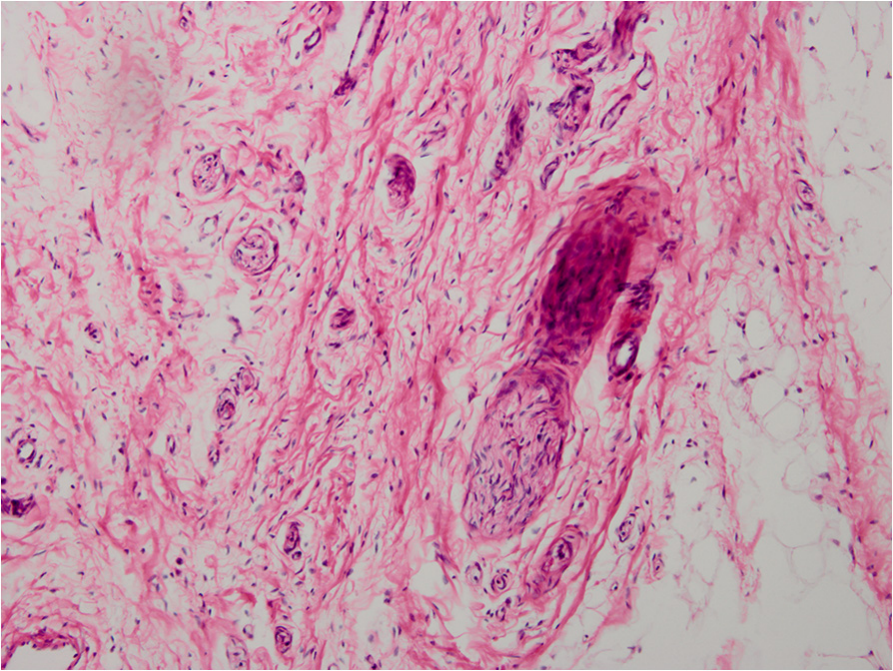
The microscopic section showed well-organized neural and bone tissue (H&E, ×200).

Laparotomy revealed a retroperitoneal membranous sac pushing the left kidney upwards and the rest of the abdominal contents to the right. The blood supply to the sac was derived from the abdominal aorta of the boy and the venous drainage was to the inferior vena cava. The sac contained muddy fluid with one fetus. On gross examination, the fetus measured 16.0 × 15.5 × 13.0 cm with a total weight of 1080.0 g. The fetus was anencephalic with malformed trunk, buttocks, intestine, and two lower limbs, of which one foot had nine toes and the other foot had four toes (Figure 2). Pathologic analysis of the resected membrane revealed that the membrane was consistent with fetal sac containing a chorionic membrane and epithelium with a marked similarity to amnion. Histopathologic examination of the fetus revealed skin with adnexa, a vertebral column with cartilage, bone and bone marrow elements, striated muscle, lymphoid tissue, sympathetic ganglia, adipose tissue, and male genitalia. Cytogenetic studies performed on the specimens showed the 46, XY karyotype (Figure 3). The boy did well and was discharged home on the 7th day postoperatively. At the last follow-up, the boy had no complaints. The serum *β*-HCG, AFP and CEA values were within normal limits.

### Case presentation N.2

**Figure 4:**
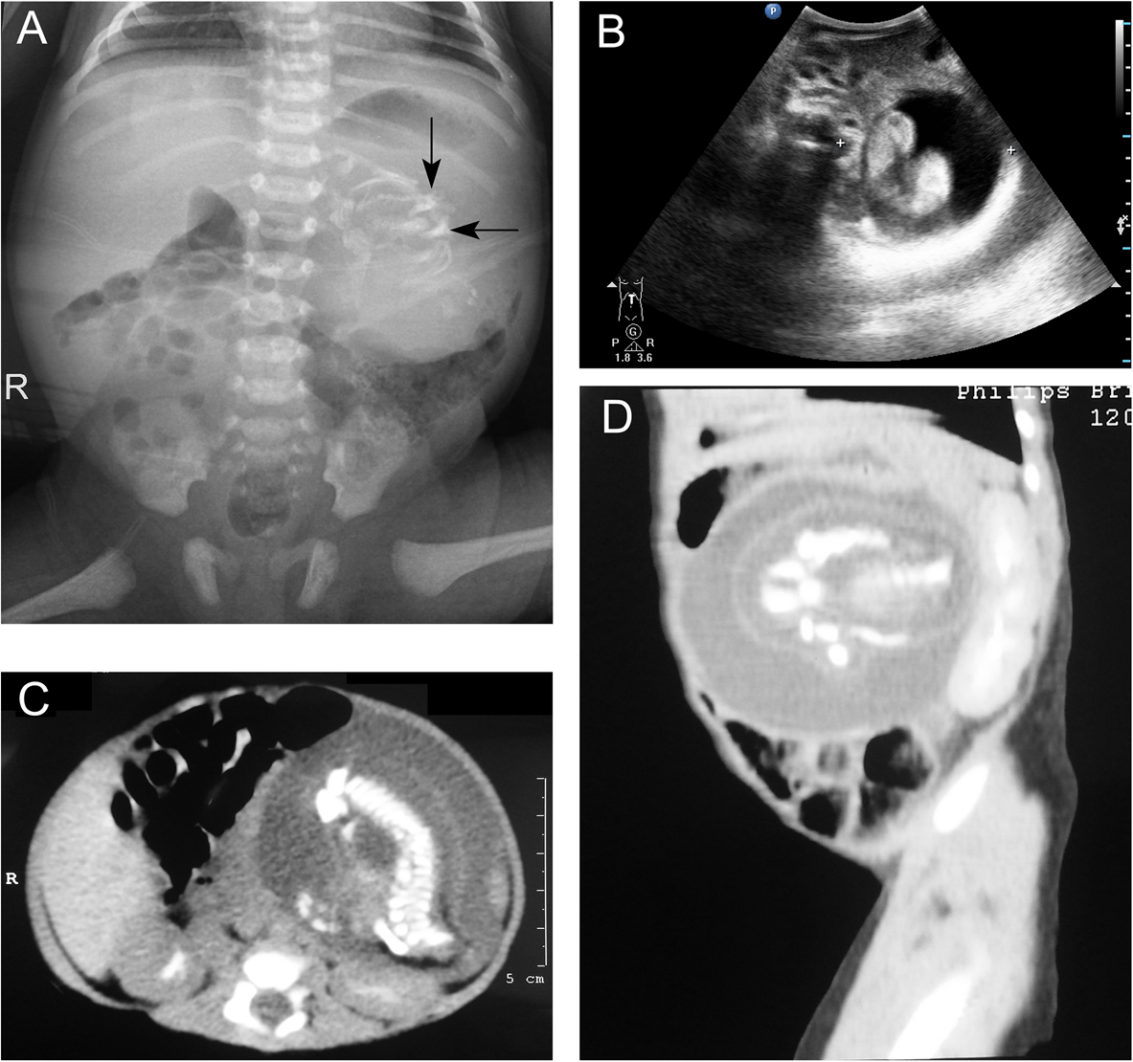
**Radiograph, ultrasonography and CT scan of the abdomen showed a large, complex, soft-tissue mass with bony elements. A**. Plain film of the abdomen. Calcified mass at the left border of thoracolumbar vertebrae T12, T11, L1 and L2. **B**. Postnatal ultrasonography of abdomen. The figure shows a retroperitoneal mass in the left upper abdomen. The mass is cystic, with a centrally located fetus. **C**. Axial CT scan at the L1 level. In front of the left kidney, there is a well-defined mass with 1 cystic formation. There are long and hyperdense opacities corresponding to fetal vertebral column. **D**. Sagittal CT scan. In front of the left kidney, there is a mass containing fluid and some calcified opacities that corresponding to fetal limbs.

A 3,660 g full-term boy was delivered by cesarean section. Apgar scores were 8 at 1 min and 9 at 5 minutes. There was no history of maternal illness, exposure to radiation, drug intake during pregnancy or consanguinity between parents. Prenatal ultrasonography performed at an outside institution at 24 weeks’ gestational age revealed a complex cystic and solid abdominal mass. On abdominal examination there was distention. A 6.0 × 5.0 cm mass was palpable in the left upper abdomen. The remainder of the exam was benign. *β*-HCG, AFP and CEA levels were within the normal ranges. The mass was confirmed by postnatal abdominal radiograph (Figure 4A). Ultrasonography (Figure 4B) and CT scan (Figure 4C) of the abdomen revealed the well encapsulated mass as retroperitoneal, located anterior to the left kidney. All three imaging study findings demonstrated bone formation. Arrangement of extremity long bones around a partially formed axial skeleton was appreciated by CT scan (Figure 4D). This finding was diagnostic of FIF.

**Figure 5:**
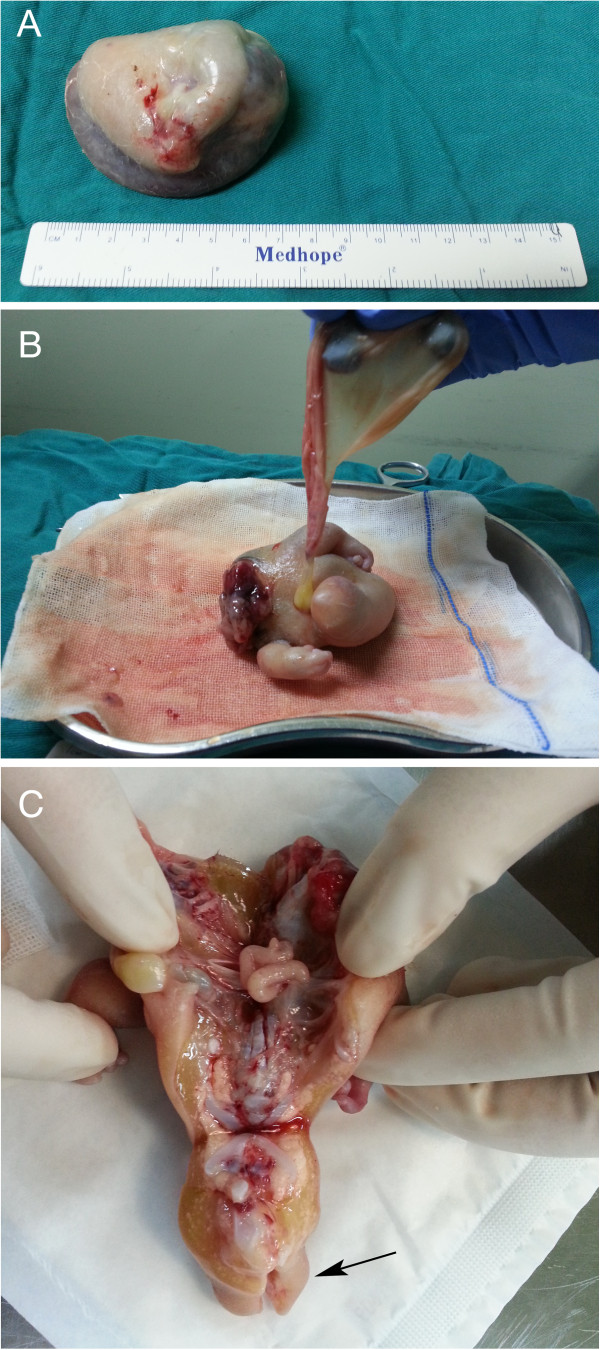
**Macroscopic view of material excised from patient 2. A**. A cystic mass covered with a membrane containing serous fluid. **B**. The postoperative specimen shows the FIF in supine position. Two upper extremities and one lower limb bud are clearly seen. **C**. Cross-section of the FIF shows a thoracic cavity, a peritoneal cavity and evidence of intestines, a spinal axis and lower limb buds.

**Figure 6:**
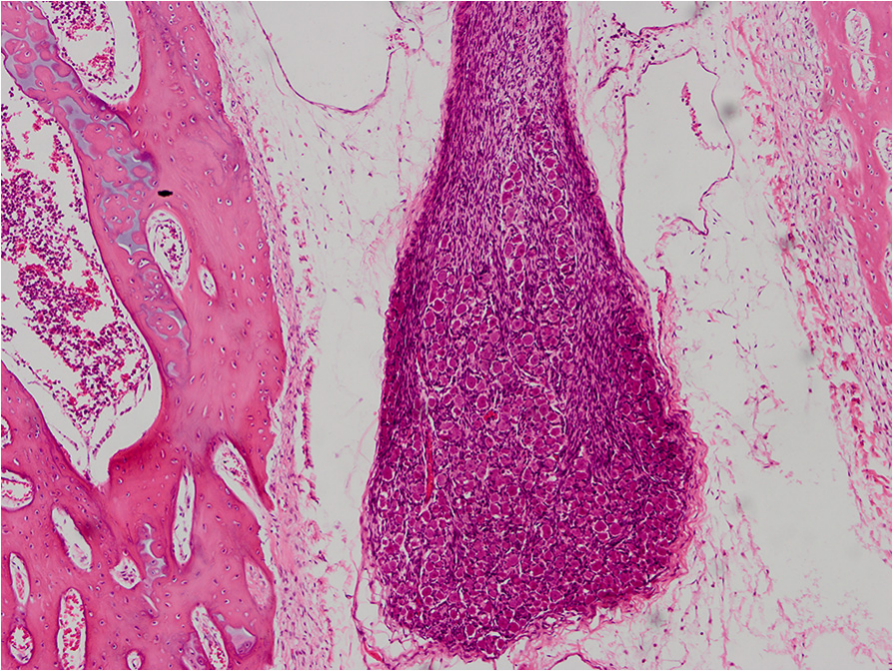
Histopathologic examination revealed neural and connective tissue (H&E, ×100).

Elective laparotomy exposed a retroperitoneal mass enveloped by a semi-transparent sac-like capsule and fed by a small branch of the splenic artery. An 8.0 × 6.0 × 6.0 cm mass was successfully resected. The capsule weighted 85.0 g and contained approximately 15 ml of serous fluid (Figure 5A). The capsule was incided and a skin-covered anencephalic FIF with a palpable vertebral column was noted. An umbilical cord-like structure was found to be contiguous with the capsule. The FIF had two upper extremities, of which one hand had five fingers and the other hand had four fingers. A lower limb bud was clearly recognizable (Figure 5B). Autopsy showed the FIF composed of thoracic cage, vertebral column and bowel-like tissue (Figure 5C, arrow). Histopathologically the FIF consisted of skin, fat, skeletal muscles and intestines. Lymph node, ganglion, nerve tissue, and peripheral nerves, bones with marrow and vertebral column with cartilage were also present (Figure 6). The karyotype of the FIF was 46 XY. The postoperative period was uneventful.

## Discussion

FIF is extremely rare pathology (1/500,000 live births) [4], in which a malformed fetus is located in the body of its twin. The liberal definition of FIF was proposed by Gonzalez-Crussi [5], who defined FIF as ‘high organotypic development and presence of a vertebral axis with arrangement of tissue around this axis’. In most cases, there is a single parasitic twin. But rarely, more than 1 parasitic twin is observed in the host body. To our knowledge, the maximum number of FIF previously documented was 11 [6]. Studies of genetic markers, such as blood group, sex chromosome constitution, protein polymorphisms, and DNA marker, suggested that host infants and their fetiform mass are genetically identical [6,7].

Eighty-nine percent of FIF lesions were noted before 18 months of ages [3]. Most FIF are located retroperitoneally along the ventral midline [8], while other rare reported sites include the cerebral ventricles [9], liver, pelvis [10], scrotum [11,12], and mediastinum [2]. Although FIF is a benign condition, the mass may compress the surrounding organs and tissue. Therefore, symptoms of FIF are primarily due to its mass effect such as abdominal distension, feeding difficulty, emesis, jaundice or pressure effects on the renal or respiratory system. Compression of the lung by the mass may explain the dyspnea in our first patient. After the surgical procedure, the boy had no further significant symptom and did well.

To qualify as an FIF, one of the following characteristics must be present: a mass enclosed within a distinct sac, partially or completed covered by skin, grossly recognizable anatomic features and attached to the host by a pedicle containing a few relatively large blood vessels [13]. The two FIFs in our report fulfilled the criteria for being FIF and not a teratoma. Ultrasonography and plain radiography can be used to achieve a diagnosis of FIF. Computed tomography scan and magnetic resonance imaging can give a more accurate diagnosis and defines the relation of the FIF with the other intra-abdominal structures [14]. In both our patients, computed tomography revealed that the mass contained irregularly shaped structures resembling a gestational sac in the middle stages of a pregnancy. The imaging played an important role in our ability to make a preoperative diagnosis.

FIF is usually overlooked in the differential diagnosis of a newborn abdominal calcification. In some cases, FIF may be confused with meconium peritonitis, which is commonly associated with calcifications [15]. Other causes of calcifications include neuroblastoma, adrenal hemorrhage, and viral infection. It is also important to differentiate between a retroperitoneal teratomas and a retroperitoneal FIF because the former have more than 10% malignancy rate. In contrast, FIF is almost always benign. Until now, only one case of malignant FIF has been reported [16]. Clinically, FIF can be differentiated from teratoma by the presence of vertebral bodies and limbs. The presence of vertebral bodies not only means that the FIF passed the primary stage of gastrulation, but also may reflect its derivation from a primitive streat. The formation of the primitive streak normally starts during the 3rd week, together with gastrulation that will lead to the notochord formation and subsequently to the vertebral column and segmental axis. Therefore, FIF likely arises from a zygote at a primitive-streak stage and fetiform mass develops to a certain degree in a manner similar to normal fetal development [7]. In contrast, teratoma consists of pluripotent cells, without organogenesis or vertebral segmentation [17]. In both our patients, pathologic examination showed vertebral column with cartilage within the mass, further supporting the diagnosis of FIF.

As described above, many authors agree that FIF corresponds to a monochorionic, monozygotic twin contained with the host [18,19]. In the study of Miura et al [7], the investigators demonstrated that host infants and their fetus shared the same genotypes, further supporting the monozygotic theory. These findings also confirmed a separate etiology for FIF as compared to teratoma. However, our knowledge of early molecular and genetic events that regulate embryo development and organogenesis is rudimentary. The possible association between FIF and highly differentiated teratoma is still controversial. Some investigators hypothesized that FIF represents a well-differentiated and highly organized teratoma [20]. In other words, FIF and teratoma may share a causal/pathogenetic mechanism. There are several observable phenomena support the teratoma theory. First, FIF are observed in the same sites as teratomas, including retroperitoneum and ovaries [1]. Second, FIF can be associated with a teratoma [21]. Retroperitoneal teratoma formation after FIF resection has also been reported [16]. A simple monozygotic (monochorionic, diamniotic) twin theory may be difficult to explain these phenomena. Third, there have been many reports of invertebrate teratomas containing well-developed fetiform structures, including brain-like tissue with ependymal-lined ‘ventricles’ and spinal cord with a central canal [22]. As there are many similarities at the histological level, and considerable overlap between FIF and teratomas, establishing the true nature of FIF is of great interesting.

The recommended treatment for FIF is surgical excision. Because the final diagnosis of FIF is not made until pathological analysis, all parts of the mass should be removed to prevent malignant recurrence. Postoperative follow-up with screening for the tumor markers *β*-HCG and AFP is often used and is further supported on the basis of malignant recurrence of FIF. The detection of raised CEA levels generally indicates advanced malignant disease. Therefore, the raised CEA level in our first patient is of great concern. Whether this association of abnormal CEA level is a manifestation of the FIF or an incidental finding is unclear based on our case. Further studies are needed to establish the significant of this phenomenon.

## Conclusions

In conclusions, the cases presented in our report meet all the accepted criteria of an abdominal FIF. The preoperative diagnosis of FIF is based on the observation of vertebral column or limbs in a mass on imaging modalities. The treatment of choice for FIF is complete resection. Future research efforts should be made to establish the true nature of FIF. Further studies to determine the possible association between FIF and highly differentiated teratoma are also warranted.

## Consent

Written informed consent for publication these case reports and accompanying images were obtained from the patients’ parents. Copies of the signed informed consent forms are available for review by the Series Editor of BMC Pediatrics.

### Ethical approval

The case reports were approved by the Ethics Committee of the West China Hospital of Sichuan University. Written informed consent was obtained for use of the images, according to the provisions of the Declaration of Helsinki.

## Abbreviations

FIF: Fetus in fetu; β-HCG: *β*-human chorionic gonadotropin; AFP: α-fetoprotein; CEA: Serum carcinoembryonic antigen.

## Competing interests

The authors declare that they have no competing interests, either financial or non-financial, that could be perceived as prejudicing the impartiality of the research reported.

## Authors’ contributions

YJ, SYC, LZ, XPJ, SGJ, FTK, QW and BX were involved in the clinical management of this patient and collected clinical details and photographs of this case report. QW and CHL collected the figures of microscopically histopathological examination. YJ and SYC reviewed the literature, and drafted the manuscript. All authors read and approved the final manuscript.

## Pre-publication history

The pre-publication history for this paper can be accessed here:

http://www.biomedcentral.com/1471-2431/14/88/prepub
